# Acute Effects of Heavy Strength Training on Mechanical, Hemodynamic, Metabolic, and Psychophysiological Parameters in Young Adult Males

**DOI:** 10.3390/sports10120195

**Published:** 2022-11-30

**Authors:** João Andrade, Dulce Esteves, Ricardo Ferraz, Diogo Luís Marques, Luís Branquinho, Daniel Almeida Marinho, Mário Cardoso Marques, Henrique Pereira Neiva

**Affiliations:** 1 Department of Sport Sciences, University of Beira Interior, Convento de Santo Atónio, 6201-001 Covilhã, Portugal; 2 Research Center in Sports Sciences, Health Sciences and Human Development, CIDESD, Convento de Santo António, 6201-001 Covilhã, Portugal; 3 Higher Institute of Educational Sciences of the Douro, 4560-708 Penafiel, Portugal

**Keywords:** strength, resistance exercise, blood pressure, heart rate, blood lactate, velocity loss, mechanical fatigue, perceived exertion

## Abstract

This study analyzed the acute effects of heavy strength training on mechanical, hemodynamic, metabolic, and psychophysiological responses in adult males. Thirteen recreational level males (23.3 ± 1.5 years) randomly performed two heavy strength training sessions (3 sets of 8 repetitions at 80% of one repetition maximum [1RM]) using the bench press (HST-BP) or full squat (HST-FS)). The repetition velocity was recorded in both sessions. Moreover, before and after the sessions, the velocity attained against the ~1.00 m·s^−1^ load (V1Load) in the HST-BP, countermovement jump (CMJ) height in the HST-FS, blood pressure, heart rate, blood lactate, and psychophysiological responses (OMNI Perceived Exertion Scale for Resistance Exercise) were measured. There were differences between exercises in the number of repetitions performed in the first and third sets (both <8 repetitions). The velocity loss was higher in the HST-BP than in the HST-FS (50.8 ± 10.0% vs. 30.7 ± 9.5%; *p* < 0.001). However, the mechanical fatigue (V1Load vs. CMJ height) and the psychophysiological response did not differ between sessions (*p* > 0.05). The HST-FS caused higher blood pressure and heart rate responses than the HST-BP (*p* < 0.001 and *p* = 0.02, respectively) and greater blood lactate changes from pre-training to post-set 1 (*p* < 0.05). These results showed that the number of maximal repetitions performed in both sessions was lower than the target number and decreased across sets. Moreover, the HST-BP caused a higher velocity loss than the HST-FS. Finally, the HST-FS elicited higher hemodynamic and metabolic demand than the HST-BP.

## 1. Introduction

Over the years, strength and conditioning coaches and researchers have proposed practical recommendations regarding manipulating the acute training variables (e.g., load or intensity, volume, exercise selection and order, movement velocity) to promote increases in muscle mass among practitioners [1,2,3,4,5]. For example, in two American College of Sports Medicine (ACSM) Position Stands, performing three sets of eight repetitions at 80% of one-repetition maximum (1RM) is within the prescription range for muscle hypertrophy in novice and intermediate practitioners [4,5]. According to the relationship between relative load (% 1RM) and the number of maximal repetitions allowed, performing eight repetitions at 80% 1RM is suggested to be equivalent to performing eight maximal repetitions (i.e., 8RM) [6]. Although it seems practical to implement the maximal repetition method into a strength training routine, some evidence suggests that the number of maximal repetitions completed against the same relative load presents high variability between individuals [7,8,9]. Consequently, prescribing the same maximal repetitions may result in different training volumes and levels of effort between individuals [7,10,11,12]. Therefore, based on these outcomes, strength and conditioning coaches and researchers have pointed out the need for alternative strategies to effectively prescribe and control the number of repetitions per set performed, such as, for example, monitoring repetition velocity during strength training [7,10,12,13,14,15,16].

Indeed, over the last decade, monitoring repetition velocity during strength training has emerged as an effective approach for prescribing training volume and relative load and quantifying neuromuscular fatigue [10,12,13,14,15,16]. In this aspect, Sánchez-Medina and González-Badillo [12] analyzed the acute mechanical and metabolic responses to different strength training designs performed to muscular failure or non-failure in strength-trained practitioners. The authors recorded each repetition velocity and quantified the mechanical fatigue through different methods, including (i) the percent velocity loss from the fastest to the slowest repetition, (ii) the percent change from pre- to post-training in the velocity attained against the ~1.00 m·s^−1^ load (V1Load), and (iii) the percent change from pre- to post-training in the countermovement jump (CMJ) height. For example, after the heavy strength training protocol of three sets of 8RM, the authors observed a velocity loss of ~40% in the full squat and ~57% in the bench press [12]. Furthermore, the higher mechanical fatigue of the heavy bench press training than the heavy full squat training was also verified in the magnitude of the V1Load changes (bench press: ~27%; full squat: ~18%) [12]. On the other hand, the heavy full squat training produced a higher metabolic demand than the bench press (i.e., higher blood lactate [La^−^] concentration) [12], thus, confirming that two different strength exercises performed with heavy loads until muscular failure cause different mechanical and metabolic responses.

In addition, it is essential to mention that 3 × 8RM protocols caused a decrease in the number of maximal repetitions performed in the bench press and full squat [7,14], thus, affecting the initially programmed training volume. Therefore, when prescribing maximal repetitions per set, it is expected to observe a decrease in the number of repetitions performed in the following sets. Furthermore, one can also speculate that the decrease in the number of repetitions per set will also contribute to a decrease in the repetition duration or the time the muscle is under tension (TUT) during exercise. According to several authors, the TUT is considered a determinant variable in optimizing muscle growth [17,18]. However, to our knowledge, no study that monitored repetition velocity has analyzed the effects of a 3 × 8RM protocol on TUT, meaning that further research is needed. The results might help understand if a significant decrease in TUT accompanies the decrease in the number of repetitions per set.

It is also essential to mention that the literature comparing the acute hemodynamic responses following a 3 × 8RM protocol in both exercises is scarce. Previous evidence with strength-trained males found that using the bench press, squat, and deadlift in the same session (i.e., 3 × 4 × 80% 1RM) decreased systolic blood pressure (SBP) after the session and 50 min later, without changes in diastolic blood pressure (DBP) [19]. Nevertheless, it remains unclear if performing only the bench press or full squat during a heavy strength training session (e.g., 3 × 8RM) produces a hypotensive effect immediately after the session. In addition, the literature is also scarce on the psychophysiological responses after heavy bench press or full squat training. Since an individual’s perceived exertion rate might help qualify the strength training session intensity [20], understanding the differences in psychophysiological responses between heavy bench press and heavy full squat training might be particularly interesting to strength coaches and researchers.

Additionally, since muscle hypertrophy is the main priority for most recreational practitioners [18], quantifying the acute hemodynamic, metabolic, mechanical, and psychophysiological responses to a heavy bench press and full squat training might be particularly relevant for the field. These findings might help strength and conditioning coaches, sport-related professionals, and researchers optimize heavy strength training prescriptions based on the type of resistance exercise, the quantity of volume, repetition duration, and relative intensity. In addition, they can give insights into the magnitude of fatigue induced by a heavy bench press and heavy full squat sessions, which might have critical applications for future study designs aiming to increase muscle size in recreational-level young adult males.

Therefore, given the above mentioned considerations, the current study aimed to analyze the acute effects of heavy bench press and full squat training on mechanical, hemodynamic, metabolic, and psychophysiological parameters in young adult males. Accordingly, the following hypotheses were defined: (i) the number of maximal repetitions completed per set and the TUT would decrease across sets; (ii) heavy bench press training would produce higher mechanical fatigue than heavy full squat training; (iii) heavy full squat training would produce a higher hemodynamic and metabolic response than heavy bench press training; (iv) no differences would be observed between training sessions in terms of psychophysiological responses.

## 2. Materials and Methods

### 2.1. Study Design

**Figure 1:**
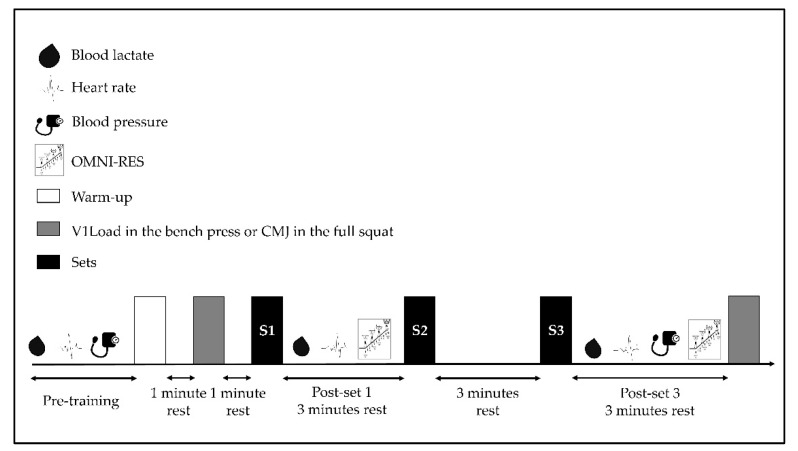
Experimental procedures of each strength training session. CMJ: countermovement jump; OMNI-RES: OMNI perceived exertion scale for resistance exercise; V1Load: velocity against the ~1.00 m·s^−1^ load.

In a crossover design, all participants performed four sessions (two per week), separated by at least 48 h. In the first week, the 1RM was estimated through a progressive loading test in the bench press and full squat. The standing height (Seca 213, Hamburg, Germany) and body composition (Tanita BC-418, Tokyo, Japan) were also measured. For standing height, the participants were measured without shoes in an upright position with the head oriented in the Frankfurt plane [21], while for body composition, they were measured without shoes. In the second week, all participants randomly performed either a heavy bench press or a full squat training constituted by three sets of 8 repetitions at 80% 1RM, following previous ACSM Position Stands [4,5]. The SBP, DBP, and heart rate (HR) were measured to analyze the hemodynamic responses. The metabolic biomarker of fatigue was the change in [La^−^]. The mechanical fatigue was assessed by analyzing the V1Load in the bench press session and the CMJ height in the full squat session. Finally, the psychophysiological response was assessed through the OMNI Perceived Exertion Scale for Resistance Exercise (OMNI-RES). All sessions were conducted in a laboratory (same time of the day) under the supervision of coaches and researchers. All participants were advised to refrain from strenuous physical exercise 48 h before each session. Figure 1 illustrates the study design.

### 2.2. Participants

Thirteen healthy young adult males (Sport Sciences students) volunteered to participate in this study. The inclusion criteria encompassed male participants aged 18 years or older, without physical limitations, familiarized with the bench press and full squat exercises performed in a Smith machine, and a training experience of at least six months. Table 1 shows the characteristics of the participants. The Ethical Committee of the University approved this study, and all participants signed written informed consent.

### 2.3. Progressive Loading Test in the Bench Press and Full Squat

Both tests were performed on a Smith machine (Multipower Fitness Line, Peroga, Murcia, Spain) coupled with a reliable linear velocity transducer (T-Force System, Ergotech, Murcia, Spain) [22]. Two spotters were positioned on each side of the barbell in both exercises to guarantee safety. In the bench press, the participants lay in a supine position with their feet on the ground, and the barbell was grasped with a pronated grip shoulder-width apart. For the full squat, the participants were in an upright position with the feet shoulder-width apart and the barbell positioned on the upper part of the trapezius. The eccentric phase was performed in a controlled manner until the barbell touched the chest (bench press) or until the posterior thighs contacted the calves (full squat) [10]. All participants were encouraged to perform the concentric phase at the maximal intended velocity without lifting the trunk during the bench press or jumping during the full squat. There was a 1–2 s pause between the eccentric and concentric phases to avoid rebound and enable more accurate measurements [23]. The initial test load was 17 kg for both exercises and increased by 10 kg after each set. In the bench press, 3 repetitions were performed for a mean propulsive velocity (MPV) > 1.00 m·s^−1^, 2 repetitions for an MPV between 0.65–1.00 m·s^−1^, and 1 repetition for an MPV < 0.65 m·s^−1^ [24]. In the full squat, 3 repetitions were performed for an MPV > 1.10 m·s^−1^, 2 repetitions for an MPV between 0.80–1.10 m·s^−1^, and 1 repetition for an MPV < 0.80 m·s^−1^ [25]. The test ended when the MPV was <0.40 m·s^−1^ and <0.60 m·s^−1^ in the bench press and full squat, respectively [10]. The inter-set rest was 3 min for 2–3 repetitions, and 5 min for one repetition. Using the last absolute load and associated MPV, the bench press 1RM load was estimated using the equation proposed by González-Badillo & Sánchez-Medina [26], while the full squat 1RM load through the equation proposed by Sánchez-Medina et al. [25].

### 2.4. Heavy Bench Press and Full Squat Training

Each training session lasted about 30 min, including warm-up and rest periods. The general warm-up included 5 min of jogging at a self-selected pace on a treadmill, plus 5 min of full-body joint mobilization, followed by a specific warm-up of 2 sets of 6 repetitions at 32% and 64% of the 1RM load, interspersed by 3 min rest [27]. After 1 min of rest, they performed the main training sets. In both sessions, an absolute load representing 80% 1RM was selected. This relative load corresponds to an MPV of ~0.48 m·s^−1^ in the bench press [26] and ~0.68 m·s^−1^ in the full squat [25]. To guarantee that all participants initiated the training according to the programmed relative load, the fastest MPV of the first set in both exercises had to match these values (±0.03 m·s^−1^). Otherwise, the absolute load (kg) had to be adjusted. After proper adjustments (when necessary), the load was maintained for all participants in the second and third sets. The target number of repetitions per set was 8, and the inter-set rest was 3 min. All participants were encouraged to displace the load at the maximal intended velocity and complete the targeted number of repetitions. The eccentric phase was performed with a controlled velocity (~2–3 s). These velocities were selected because slow eccentric and moderate to fast concentric repetitions may benefit muscle hypertrophy [3]. After both sessions, the following variables were analyzed for each set and the three sets (i.e., the average of the three sets): the number of repetitions, TUT (calculated as the sum of the total time (in seconds, s) of the concentric and eccentric phases in each set [28]), fastest MPV (MPV_fast_), slowest MPV (MPV_slow_), average MPV (MPV_avg_), and velocity loss (calculated as the percent loss in MPV from the fastest to the slowest repetition [7]).

### 2.5. Hemodynamic and Metabolic Measures

The first evaluations of SBP, DBP, HR, and [La^−^] were made before warm-up and after a 5-min rest in a chair. SBP and DBP were measured immediately after set 3. The same procedure was done for HR and [La^−^], but with an additional measure after set 1. We used an automatic blood pressure monitor (Omron HEM-7113 model, Kyoto, Japan) to measure SBP and DBP with the cuff size adapted to each participant’s arm circumference [29]. An HR monitor (Polar H10, Kempele, Finland) connected via Bluetooth to a Polar watch attached below the chest was used to monitor HR. For [La^-^] measures, after cleansing the site with 70% alcohol, each participant’s fingertip was punctured using a disposable lancet. The first drop of blood was discarded to avoid contamination with sweat, and then a tiny blood sample was collected for analysis (Lactate Pro 2 LT-1730, Tokyo, Japan) [29]. The absolute values and the percent change throughout measures were used for analysis.

### 2.6. Mechanical Fatigue and Psychophysiological Measures

After the warm-up and 3 min after the third set, we measured the V1Load in the bench press session and the CMJ height in the full squat session. Previous researchers have already used these variables to quantify mechanical fatigue after heavy strength training sessions [12,14,15]. All participants performed three repetitions in the bench press at the maximal intended velocity against the ~1.00 m·s^−1^ load. The V1Load was previously identified during the progressive loading test. For the CMJ, all participants started from an upright position with arms akimbo and feet shoulder-width apart, and performed a rapid downward movement (~90° knee flexion) immediately followed by a maximal vertical jump. Three CMJs were performed, and the height was estimated using an infrared timing system (Optojump, Microgate, Bolzano, Italy). The maximum values were kept for analysis. The psychophysiological response was determined by the OMNI-RES, which is a qualitative measure of the effort that presents both verbal and mode-specific pictorial descriptors distributed through a range of 0 (extremely easy) to 10 (extremely hard) [20]. The OMNI-RES values were obtained after sets 1 and 3.

### 2.7. Statistical Analysis

Data are presented as mean ± SD unless otherwise indicated. The normality and homogeneity of variances were analyzed using the Shapiro-Wilk and Levene tests, respectively. A 3 × 2 repeated-measures ANOVA (within subject factor: 3 sets; between subject factor: 2 exercises) with post hoc Bonferroni tests analyzed the effects of the bench press and full squat sessions across the three training sets on the acute training variables (i.e., number of repetitions, TUT, MPV_fast_, MPV_slow_, MPV_avg_, and velocity loss). An independent samples t-test compared the differences between exercises in the average of the three sets in each variable and the percent change (%Δ = ([post-session − pre-session]/pre-session) × 100) in SBP, DBP, HR, [La^−^], V1Load, CMJ, and OMNI-RES. In addition, paired samples *t*-tests examined the differences from pre- to post-sessions on hemodynamic, metabolic, and psychophysiological variables. The effect size was calculated using the Hedge’s *g* and interpreted as follows: trivial (*g* < 0.2), small (*g* = 0.2–0.6), moderate (*g* = 0.6–1.2), large (*g* = 1.2–2.0), very large (*g* = 2.0–4.0), and extremely large (*g* > 4.0) [30]. The significance level was set at *p* < 0.05. Statistical data were analyzed using Microsoft Office Excel^®^ (Microsoft Inc., Redmond, WA, USA) and SPSS v28 (SPSS Inc., Chicago, IL, USA). The figures were designed in GraphPad Prism v7 (GraphPad Inc., San Diego, CA, USA).

## 3. Results

### 3.1. Characteristics of the Heavy Bench Press and Full Squat Training Sessions

Table 2 shows the effects of the bench press and full squat across the training sets on the acute training variables. The number of repetitions per set performed in both exercises was lower than the target number (i.e., eight repetitions). In the bench press, the number of repetitions decreased from the first to the third set, while in the full squat, it decreased from the first to the second set. There were differences between exercises in the number of repetitions performed in the first and third sets. The TUT decreased from the first to the third set in the bench press. However, there were no differences between resistance exercises and sets in the TUT. Regarding repetition velocity, the MPV_fast_ of the first set was ~0.47 m·s^−1^ in the bench press and ~0.68 m·s^−1^ in the full squat, confirming that all participants started the session according to the programmed relative load of 80% 1RM [25,26]. There was a decrease in the MPV_fast_ from the first and second sets to the third set in the bench press, and a decrease from the first to the second and third sets in the full squat. In addition, there was a decrease in the MPV_avg_ from the first and second sets to the third set in the full squat. Regarding velocity loss, there was a decrease from the second to the third set in the bench press. Finally, there were large to extremely large differences between exercises in all velocity variables in all training sets.

### 3.2. Acute Hemodynamic, Metabolic, Mechanical, and Psychophysiological Responses after Sessions

Table 3 shows the acute hemodynamic, metabolic, mechanical, and psychophysiological responses after sessions. Although both sessions significantly increased the HR and [La^−^] after training, only the full squat significantly increased the SBP and DBP. Regarding the mechanical responses, the bench press session decreased the V1Load after training, and the full squat decreased the CMJ height from pre- to post-training. There was a significant increase in OMNI-RES from the first to the third set in both sessions, corresponding to a hard and extremely hard rating.

### 3.3. Comparison between Sessions on Hemodynamic, Metabolic, Mechanical, and Psychophysiological Changes

**Figure 2:**
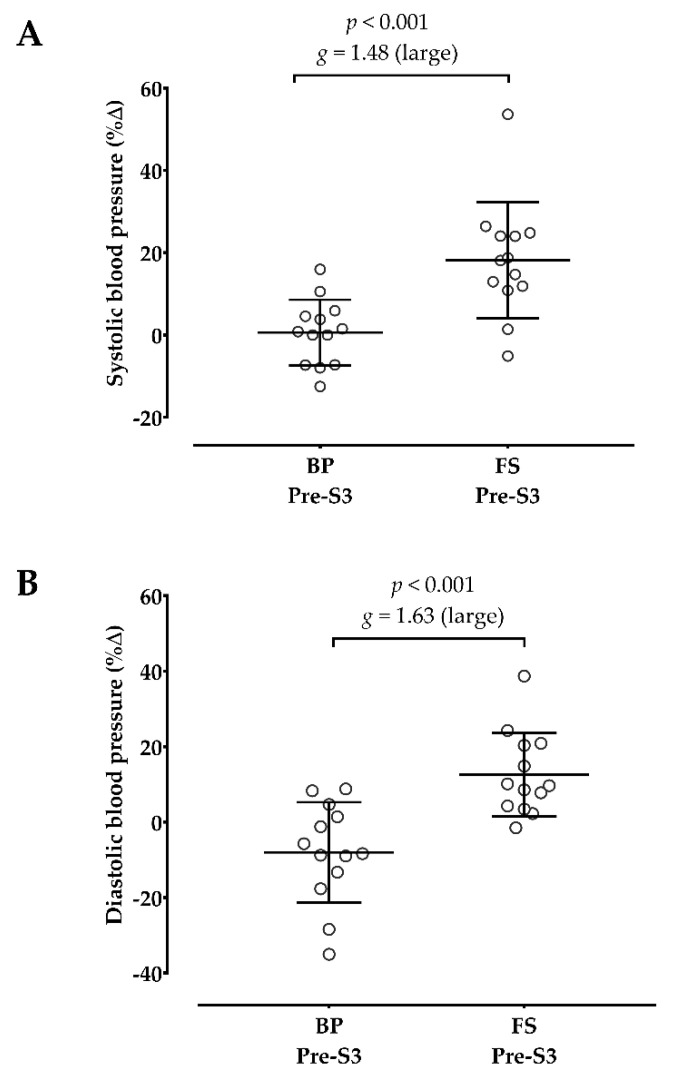
Comparison between the bench press (BP) and full squat (FS) in systolic (**A**) and diastolic blood pressure (**B**) changes. %Δ: percent change; *g*: Hedge’s g effect size; S3: post-set 3. The open circles represent the individuals’ changes.

Figure 2 shows that the full squat produced larger SBP and DBP changes from pre- to post-session than the bench press.

**Figure 3:**
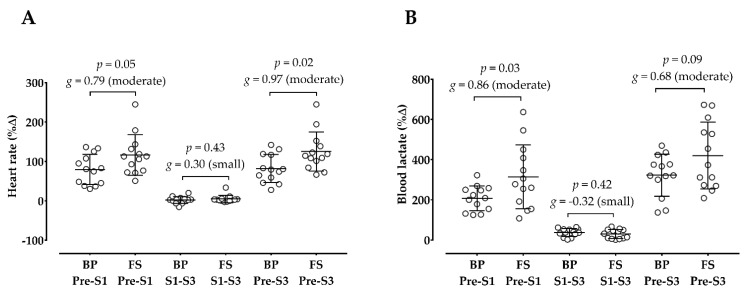
Comparison between the bench press (BP) and full squat (FS) in heart rate (**A**) and blood lactate (**B**) changes. %Δ: percent change; *g*: Hedge’s *g* effect size; S1: post-set 1; S3: post-set 3. The open circles represent the individuals’ changes.

Figure 3 demonstrates that the full squat produced a greater HR change from pre-training to post-set 3 than the bench press. In addition, Figure 3 shows that the full squat produced a greater [La^−^] change from pre-training to post-set 1 than the bench press, but from pre-training to post-set 3 there were no differences between sessions on [La^−^] change.

**Figure 4:**
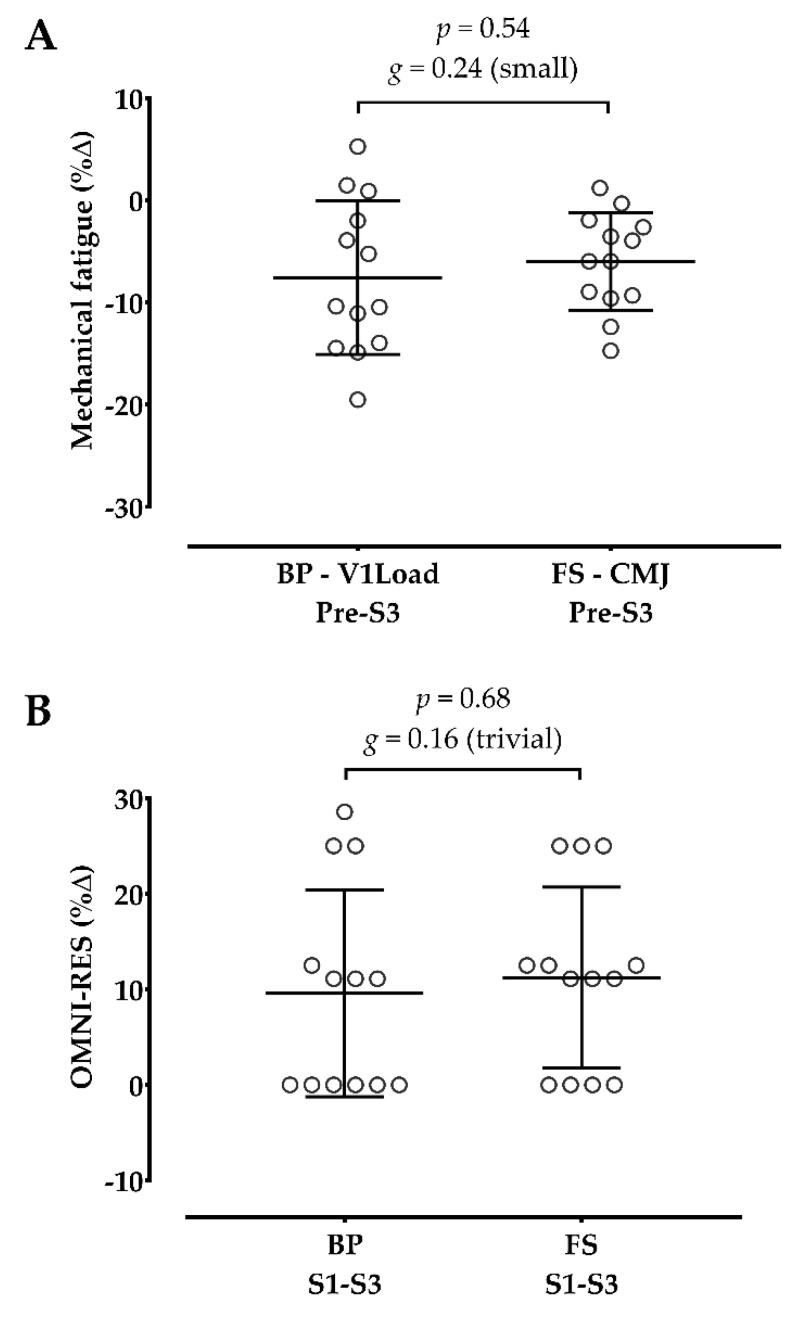
Comparison between the bench press (BP) and full squat (FS) in mechanical fatigue (**A**) and psychophysiological (**B**) changes. %Δ: percent change; *g*: Hedge’s *g* effect size; S1: post-set 1; S3: post-set 3; V1Load: mean propulsive velocity achieved with the load that allowed to reach ~1.00 m·s^−1^; CMJ: countermovement jump; OMNI-RES: rate of perceived exertion scale. The open circles represent the individuals’ changes.

Figure 4 shows no differences between sessions on mechanical fatigue (V1Load vs. CMJ) and psychophysiological (i.e., OMNI-RES) changes.

## 4. Discussion

### 4.1. Main Findings

The current study compared the acute mechanical, hemodynamic, metabolic, and psychophysiological responses to a heavy bench press or full squat training in recreational-level young adult males. The main findings were: (i) the number of maximal repetitions performed per set in both sessions was lower than the target number; (ii) the number of maximal repetitions and the TUT decreased across sets in both sessions; (iii) the bench press caused a higher velocity loss than the full squat; (iv) both sessions decreased mechanical performance and increased the psychophysiological responses, without differences between sessions in both parameters; (v) the full squat caused a higher acute hemodynamic and metabolic response than the bench press.

### 4.2. Acute Effects of Heavy Bench Press and Full Squat on the Number of Repetitions and Time under Tension

Following the proposed protocol by ACSM for muscle hypertrophy [4,5], it was observed that the number of repetitions performed per set in both strength training sessions was lower than the target number (i.e., eight). In addition, the completed number of repetitions per set varied between participants, which agrees with previous studies that found similar variability after a heavy strength session (i.e., 3 × 8RM) [7,14]. Interestingly, in the current study, the total repetitions performed in both exercises were even lower (2 repetitions less in the bench press and 1 in the full squat) than those reported in previous studies (bench press: 7.6 ± 0.6; full squat: 7.4 ± 0.7) [7,14]. Therefore, these data suggest that even for practitioners with more than six months of strength training background, training sets of 8RM will result in fewer repetitions than stipulated.

Importantly, the current study observed that the number of maximal repetitions completed tended to decrease across sets, with greater sustainability of repetitions for the full squat than the bench press, as likely observed in previous research [31]. Previous findings also showed that when performing 8–15RM in the first set, either in the bench press or back squat, the number of repetitions decreases in the following sets, especially when the recovery time between sets is 1–3 min [31,32,33]. As several authors mentioned, the inability to complete the same number of maximal repetitions in the following sets may result from the lingering effects of hydrogen ions accumulation in recreational-level males [33]. Therefore, increasing the inter-set rest from three to five minutes may be needed to maintain the capacity to perform the requested number of repetitions in the following sets, as this time seems indicated to recover intramuscular adenosine triphosphate [ATP] and phosphocreatine [PCr], eliminate hydrogen ions, and restore force production in recreational level males [31,32,33]. As opposed, highly strength trained individuals, especially bodybuilders, might require only 1–2 min of inter-set rest to restore energy sources and perform the same number of maximal repetitions in the following sets [32]. The higher capacity of bodybuilders to resist mechanical fatigue is thought to be due to their training routines, which usually consist of moderate to high volume and short inter-set rests [31]. Therefore, when prescribing a heavy strength training session, it is essential to understand that training status is a determining factor in successfully performing a 3 × 8 × 80% 1RM session.

Additionally, there was a decrease in TUT across sets in both sessions, possibly due to the previous physiological explanations. Although the decrease in TUT was more pronounced in the bench press than the full squat, particularly from the first to the third set, both exercises experienced a decrease right after the first set. Therefore, this information highlights that performing maximal repetitions per set will also affect the TUT and change the configuration of the initially programmed training stimulus in recreational-level males.

### 4.3. Acute Mechanical Responses

According to Sánchez-Medina and González-Badillo [12], measuring the V1Load and CMJ height changes from pre- to post-training are two reliable methods for quantifying mechanical fatigue, as they are related to the loss of muscle-shortening velocity. In addition, the V1Load and CMJ height loss are strongly associated with [La^−^] and ammonia [12], thus, assuming their relevance for quantifying muscle fatigue. In the current study, as expected, the bench press and full squat significantly decreased mechanical performance after training sessions, which is in line with previous reports [10,12,14,34]. Furthermore, although the current study did not analyze the time course of recovery following exercise, it is worthwhile to mention that previous research observed that the initial values of V1Load and CMJ height were not fully restored after 48 h of performing the 3 × 8RM protocol [14,15]. In addition, previous data indicated that a 3 × 8RM protocol contributed to greater plasma creatine kinase (CK) levels than a non-failure protocol [15]. Therefore, as CK is a delayed fatigue marker [16], these findings reinforce that a 3 × 8RM protocol leads to high mechanical fatigue and low post-exercise recovery.

Another data that needs to be addressed is that although mechanical fatigue changes were not different between exercises (maybe because of the variables chosen for analysis: V1Load vs. CMJ), the velocity loss was higher in the bench press than in the full squat, which is supported by previous studies [10,12,13,14,15,34]. These differences might be associated with the smaller muscle groups recruited during the bench press than the full squat and a higher percentage of fast-twitch fibers in the upper than the lower musculature, contributing to the early appearance of fatigue [10,12]. Consequently, prescribing the same training in the bench press and full squat (i.e., 3 × 8 × 80% 1RM) for all practitioners will produce different levels of neuromuscular fatigue in the upper and lower limbs.

### 4.4. Acute Hemodynamic and Metabolic Responses

As expected, the current results demonstrated that the full squat produced a higher acute hemodynamic response than the bench press. This occurrence might be associated with more muscle mass required during the full squat than the bench press. According to several authors, the higher the recruitment of muscle mass, the more occlusion, which results in high blood pressure and reduced blood flow [35]. Despite the significant increase in HR after the bench press session, the SBP and DBP did not change. Research with young adults observed that 15 min after a heavy strength training session (3 × 10 × 80% 1RM), HR increased, and the SBP and DBP did not change, which agrees with our data despite the time difference in the measurements [36]. Also, considering that the supine position increases blood flow in the upper body [37], this factor might have masked an eventual hypotensive effect after the bench press. Regarding [La^−^], the full squat produced a significantly higher metabolic increase than the bench press from pre-training to post-set 1. These results were already observed after heavy strength training sessions (3 × 8RM) [12], thus reinforcing its higher metabolic demand.

### 4.5. Acute Psychophysiological Responses

The results demonstrated that both sessions were classified between hard and extremely hard, thus, reflecting the high psychophysiological response of heavy strength training. Nevertheless, despite the significant increase in OMNI-RES from sets 1 to 3, the changes produced from the first to the third set were not different between exercises, confirming a similar psychophysiological response between the bench press and full squat.

### 4.6. Limitations

This study has some limitations. First, a larger sample size of male and female participants with different strength training backgrounds would be interesting to analyze how sex and training experience influence the responses. Second, additional strength training protocols with different volume and relative intensity configurations would allow us to compare the magnitude of fatigue between different sessions and further understand the current results. Finally, examining the time course of recovery between sessions would help understand the optimal recovery periods.

## 5. Conclusions

The current study showed that recreational level young adult males could not perform the maximal number of repetitions per set requested by a traditional heavy strength training protocol for muscle hypertrophy (3 × 8 × 80% 1RM). In addition, the number of maximal repetitions performed and the TUT during exercise decreased across sets in both exercises, meaning that the actual training performed deviated from the intended one. Furthermore, although no differences were found between sessions on mechanical fatigue and psychophysiological responses, the heavy bench press caused a significantly higher velocity loss than the heavy full squat, suggesting higher neuromuscular fatigue of the former exercise [12]. In this sense, caution should be taken by strength coaches and researchers when prescribing maximal repetitions, especially for naïve practitioners, due to a potential decrease in repetitions performed and TUT across sets and high mechanical fatigue, which may delay recovery and affect the intended muscular adaptations. As an alternative, increasing the recovery time between sets from three to five minutes might be a practical solution to restore energy sources and maintain the ability to perform the requested maximal number of repetitions in recreational-level young adult males.

The results of this research also highlight that the heavy full squat caused higher hemodynamic and metabolic responses than the heavy bench press, confirming a higher cardiovascular demand of the full squat. Therefore, the results presented here highlight the importance of individualizing the training based on the type of resistance exercise and monitoring the acute physical, physiological, and psychophysiological responses to objectively control the training load (e.g., volume and relative intensity), adjust it in real-time whenever needed, and quantify the degree of fatigue in young adult males.

## Figures and Tables

**Table 1:** Participants’ characteristics.

Variable	Mean ± SD	95% Confidence Interval
Age	23.3 ± 1.5	22.5–24.1
Body mass (kg)	80.5 ± 8.7	75.8–85.2
Standing height (cm)	175.9 ± 3.4	174.1–177.8
Body Mass Index (kg/m^2^)	26.0 ± 2.7	24.6–27.4
Fat Mass (%)	18.8 ± 5.0	16.1–21.6
Muscle Mass (kg)	65.0 ± 4.1	62.7–67.2
Basal Metabolic Rate (calories)	1932.3 ± 132.1	1860.5–2004.1
Systolic Blood Pressure (mmHg)	132.4 ± 9.4	127.3–137.5
Diastolic Blood Pressure (mmHg)	73.2 ± 10.0	67.8–78.7
Heart Rate Rest (bpm)	69.1 ± 12.7	62.2–76.0
Strength Training Experience (years)	1.4 ± 1.4	0.7–2.2
1RM Bench Press (kg)	75.7 ± 9.5	70.5–80.8
Relative 1RM Bench Press (kg/body mass)	0.95 ± 0.17	0.86–1.05
1RM Full Squat (kg)	87.9 ± 16.4	79.0–96.8
Relative 1RM Full Squat (kg/body mass)	1.11 ± 0.26	0.97–1.25

RM: repetition maximum.

**Table 2:** Effects of the bench press vs. full squat across the training sets on the acute training variables.

		Set 1	Set 2	Set 3	All *	Set 1 vs. Set 2		Set 1 vs. Set 3		Set 2 vs. Set 3	
Acute Variable	Session	Mean ± SD	Mean ± SD	Mean ± SD	Mean ± SD	*p*-Value	*g*	*p*-Value	*g*	*p*-Value	*g*
Repetitions	BP	5.9 ± 1.9	5.8 ± 1.8	4.6 ± 1.9	5.5 ± 1.7	1.00	−0.04	**0.03**	−0.65	0.08	−0.63
	FS	7.3 ± 1.0	6.2 ± 1.7	6.2 ± 1.9	6.6 ± 1.2	**0.01**	−0.71	0.09	−0.67	1.00	0.00
	BP vs. FS	***p*** ** = 0.03**	*p* = 0.59	***p*** ** = 0.04**	*p* = 0.60						
		*g =* −0.88	*g =* −0.21	*g =* −0.84	*g =* −0.75						
TUT (s)	BP	16.4 ± 5.0	16.2 ± 5.2	12.9 ± 4.2	15.2 ± 4.4	1.00	−0.03	**0.02**	−0.69	0.08	−0.63
	FS	17.6 ± 5.0	15.3 ± 4.1	15.6 ± 4.6	16.2 ± 3.7	0.09	−0.46	0.30	−0.39	1.00	0.05
	BP vs. FS	*p* = 0.56	*p* = 0.64	*p* = 0.14	*p* = 0.54						
		*g =* −0.23	*g =* 0.18	*g =* −0.57	*g =* −0.23						
MPV_fast_ (m·s^−1^)	BP	0.47 ± 0.03	0.46 ± 0.04	0.42 ± 0.07	0.45 ± 0.03	1.00	−0.24	**0.03**	−1.00	**0.04**	−0.75
	FS	0.68 ± 0.03	0.63 ± 0.08	0.60 ± 0.08	0.64 ± 0.05	**0.01**	−0.85	**<0.001**	−1.34	0.24	−0.38
	BP vs. FS	***p* < ** **0.001**	***p* < ** **0.001**	***p* < ** **0.001**	***p* < ** **0.001**						
		*g =* −7.54	*g =* −2.62	*g =* −2.45	*g =* −4.16						
MPV_slow_ (m·s^−1^)	BP	0.22 ± 0.06	0.21 ± 0.07	0.23 ± 0.05	0.22 ± 0.05	1.00	−0.12	1.00	0.28	0.13	0.40
	FS	0.45 ± 0.09	0.44 ± 0.08	0.42 ± 0.09	0.44 ± 0.08	1.00	−0.06	0.60	−0.28	0.28	−0.22
	BP vs. FS	***p* < ** **0.001**	***p* < ** **0.001**	***p* < ** **0.001**	***p* < ** **0.001**						
		*g =* −2.97	*g =* −3.01	*g =* −2.37	*g =* −3.12						
MPV_avg_ (m·s^−1^)	BP	0.35 ± 0.03	0.34 ± 0.04	0.33 ± 0.06	0.34 ± 0.04	1.00	−0.27	0.44	−0.43	0.93	−0.17
	FS	0.55 ± 0.06	0.54 ± 0.07	0.51 ± 0.07	0.53 ± 0.06	0.49	−0.24	**0.02**	−0.60	**0.03**	−0.32
	BP vs. FS	***p* < ** **0.001**	***p* < ** **0.001**	***p* < ** **0.001**	***p* < ** **0.001**						
		*g =* −4.20	*g =* −3.16	*g =* −2.79	*g =* −3.62						
Velocity loss (%)	BP	53.9 ± 13.6	54.8 ± 13.9	43.6 ± 9.7	50.8 ± 10.0	1.00	0.06	0.06	−0.82	**0.01**	−0.88
	FS	34.0 ± 11.2	29.1 ± 10.6	28.9 ± 14.1	30.7 ± 9.5	0.38	−0.42	0.69	−0.37	1.00	−0.01
	BP vs. FS	***p* < ** **0.001**	***p* < ** **0.001**	***p* < ** **0.01**	***p* < ** **0.001**						
		*g =* 1.55	*g =* 2.01	*g =* 1.17	*g =* 1.99						

Significant differences are marked in bold; * The “All” variable corresponds to the average of the three sets; SD: standard deviation; BP: bench press; FS: full squat; TUT: time under tension; *p:* differences between sets and exercises; *g*: Hedge’s g effect size; MVP_fast_: fastest mean propulsive velocity; MVP_slow_: slowest mean propulsive velocity; MVP_avg_: average mean propulsive velocity.

**Table 3:** Acute hemodynamic, metabolic, mechanical, and perceived exertion responses from pre- to post-training in the bench press and full squat training.

Session	Variable	Pre-Training	Post-Training	Mean Difference	*p*-Value	*g*	%Δ (90% CI)
BP	SBP (mmHg)	132.1 ± 10.3	132.4 ± 8.0	0.3 ± 10.3	0.92	0.03	0.6 (−3.0; 4.3)
	DBP (mmHg)	70.15 ± 9.2	64.0 ± 9.4	−6.2 ± 10.2	0.05	−0.64	−8.0 (−14.1; −1.9)
	HR (bpm)	76.3 ± 14.5	134.92 ± 18.15	58.6 ± 18.9	**<0.001**	3.46	81.8 (65.6; 98.1)
	[La^−^] (mmol/L)	1.4 ± 0.3	5.7 ± 1.7	4.3 ± 1.6	**<0.001**	3.38	322.8 (275.0; 370.5)
	V1Load (m·s^−1^)	1.01 ± 0.02	0.93 ± 0.08	−0.08 ± 0.08	**<0.01**	−1.30	−7.6 (−11.0; −4.1)
	OMNI-RES *	8.4 ± 0.9	9.15 ± 1.0	0.8 ± 0.8	**<0.01**	0.80	9.6 (4.6; 14.5)
FS	SBP (mmHg)	133.5 ± 8.0	157.3 ± 16.8	23.8 ± 17.8	**<0.001**	1.76	18.2 (11.8; 24.6)
	DBP (mmHg)	70.9 ± 8.8	79.4 ± 8.3	8.5 ± 6.9	**<0.001**	0.96	12.6 (7.6; 17.6)
	HR (bpm)	73.2 ± 12.9	159.2 ± 10.6	86.1 ± 16.6	**<0.001**	7.06	125.1 (102.6; 147.6)
	[La^−^] (mmol/L)	1.4 ± 0.3	7.2 ± 2.5	5.8 ± 2.4	**<0.001**	3.13	420.1 (344.1; 496.1)
	CMJ (cm)	31.1 ± 3.7	29.2 ± 3.8	−1.9 ± 1.5	**<0.001**	−0.48	−6.0 (−8.2; −3.8)
	OMNI-RES *	8.5 ± 0.7	9.5 ± 0.7	0.9 ± 0.8	**<0.001**	1.35	11.2 (6.9; 15.5)

Significant differences are marked in bold; BP: bench press: FS: full squat; %Δ: percent change; CI: confidence interval; *g*: Hedge’s g effect size; SBP: systolic blood pressure; DBP: diastolic blood pressure; HR: heart rate; [La^−^]: blood lactate; V1Load: mean propulsive velocity achieved with the load that allowed to reach ~1.00 m·s^−1^; OMNI-RES: rate of perceived exertion scale; CMJ: countermovement jump; * data obtained after sets 1 and 3.

## Data Availability

The data presented in this study are available on request from the corresponding author.

## References

[1] Kraemer W.J., Ratamess N.A. (2004). Fundamentals of resistance training: Progression and exercise prescription. Med. Sci. Sports Exerc..

[2] Shimano T., Kraemer W.J., Spiering B.A., Volek J.S., Hatfield D.L., Silvestre R., Vingren J.L., Fragala M.S., Maresh C.M., Fleck S.J. (2006). Relationship between the number of repetitions and selected percentages of one repetition maximum in free weight exercises in trained and untrained men. J. Strength Cond. Res..

[3] Schoenfeld B.J. (2010). The Mechanisms of Muscle Hypertrophy and Their Application to Resistance Training. J. Strength Cond. Res..

[4] Ratamess N.A., Alvar B.A., Evetoch T.K., Housh T.J., Kibler W.B., Kraemer W.J., Triplett N.T. (2009). Progression Models in Resistance Training for Healthy Adults. Med. Sci. Sports Exerc..

[5] Kraemer W.J., Adams K., Cafarelli E., Dudley G.A., Dooly C., Feigenbaum M.S., Fleck S.J., Franklin B., Fry A.C., Hoffman J.R. (2002). Progression Models in Resistance Training for Healthy Adults. Med. Sci. Sports Exerc..

[6] Sheppard J.M., Triplett N.T., Haff G.G., Triplett N.T. (2016). Program Design for Resistance Training. Essentials of Strength Training and Conditioning.

[7] González-Badillo J.J., Yañez-Garcia J.M., Mora-Custodio R., Rodríguez-Rosell D. (2017). Velocity Loss as a Variable for Monitoring Resistance Exercise. Int. J. Sports Med..

[8] Richens B., Cleather D.J. (2014). The relationship between the number of repetitions performed at given intensities is different in endurance and strength trained athletes. Biol. Sport.

[9] Terzis G., Spengos K., Manta P., Sarris N., Georgiadis G. (2008). Fiber Type Composition and Capillary Density in Relation to Submaximal Number of Repetitions in Resistance Exercise. J. Strength Cond. Res..

[10] Rodríguez-Rosell D., Yanez-Garcia J.M., Torres-Torrelo J., Mora-Custodio R., Marques M.C., González-Badillo J.J. (2018). Effort Index as a Novel Variable for Monitoring the Level of Effort During Resistance Exercises. J. Strength Cond. Res..

[11] Pareja-Blanco F., Villalba-Fernández A., Cornejo-Daza P.J., Sánchez-Valdepeñas J., González-Badillo J.J. (2019). Time Course of Recovery Following Resistance Exercise with Different Loading Magnitudes and Velocity Loss in the Set. Sports.

[12] Sánchez-Medina L., González-Badillo J. (2011). Velocity loss as an indicator of neuromuscular fatigue during resistance training. Med. Sci. Sports Exerc..

[13] Pareja-Blanco F., Rodríguez-Rosell D., Sánchez-Medina L., Ribas-Serna J., López-López C., Mora-Custodio R., Yáñez-García J.M., González-Badillo J.J. (2017). Acute and delayed response to resistance exercise leading or not leading to muscle failure. Clin. Physiol. Funct. Imaging.

[14] Pareja-Blanco F., Rodríguez-Rosell D., Aagaard P., Sánchez-Medina L., Ribas-Serna J., Mora-Custodio R., Otero-Esquina C., Yáñez-García J.M., González-Badillo J.J. (2018). Time Course of Recovery From Resistance Exercise With Different Set Configurations. J. Strength Cond. Res..

[15] González-Badillo J.J., Rodríguez-Rosell D., Sánchez-Medina L., Ribas J., López-López C., Mora-Custodio R., Yañez-García J.M., Pareja-Blanco F. (2016). Short-term Recovery Following Resistance Exercise Leading or not to Failure. Int. J. Sports Med..

[16] Morán-Navarro R., Pérez C.E., Mora-Rodríguez R., de la Cruz-Sánchez E., González-Badillo J.J., Sánchez-Medina L., Pallarés J.G. (2017). Time course of recovery following resistance training leading or not to failure. Eur. J. Appl. Physiol..

[17] Burd N.A., Andrews R.J., West D.W.D., Little J.P., Cochran A.J.R., Hector A.J., Cashaback J.G.A., Gibala M.J., Potvin J.R., Baker S.K. (2012). Muscle time under tension during resistance exercise stimulates differential muscle protein sub-fractional synthetic responses in men. J. Physiol..

[18] Krzysztofik M., Wilk M., Wojdała G., Gołaś A. (2019). Maximizing Muscle Hypertrophy: A Systematic Review of Advanced Resistance Training Techniques and Methods. Int. J. Environ. Res. Public Health.

[19] Duncan M.J., Birch S.L., Oxford S.W. (2014). The Effect of Exercise Intensity on Postresistance Exercise Hypotension in Trained Men. J. Strength Cond. Res..

[20] Lagally K.M., Robertson R.J. (2006). Construct Validity if the OMNI Resistance Exercise Scale. J. Strength Cond. Res..

[21] Rinaldo N., Toselli S., Gualdi-Russo E., Zedda N., Zaccagni L. (2020). Effects of Anthropometric Growth and Basketball Experience on Physical Performance in Pre-Adolescent Male Players. Int. J. Environ. Res. Public Health.

[22] Courel-Ibáñez J., Martínez-Cava A., Morán-Navarro R., Escribano-Peñas P., Chavarren-Cabrero J., González-Badillo J.J., Pallarés J.G. (2019). Reproducibility and Repeatability of Five Different Technologies for Bar Velocity Measurement in Resistance Training. Ann. Biomed. Eng..

[23] Pallarés J.G., Sánchez-Medina L., Pérez C.E., De La Cruz-Sánchez E., Mora-Rodriguez R. (2014). Imposing a pause between the eccentric and concentric phases increases the reliability of isoinertial strength assessments. J. Sports Sci..

[24] Sánchez-Medina L., Pérez C.E., González-Badillo J.J. (2010). Importance of the Propulsive Phase in Strength Assessment. Int. J. Sports Med..

[25] Sánchez-Medina L., Pallarés J.G., Pérez C.E., Morán-Navarro R., González-Badillo J.J. (2017). Estimation of Relative Load From Bar Velocity in the Full Back Squat Exercise. Sports Med. Int. Open.

[26] González-Badillo J.J., Sánchez-Medina L. (2010). Movement Velocity as a Measure of Loading Intensity in Resistance Training. Int. J. Sports Med..

[27] Ribeiro B., Pereira A., Neves P.P., Sousa A.C., Ferraz R., Marques M.C., Marinho D.A., Neiva H.P. (2020). The role of specific warm-up during bench press and squat exercises: A novel approach. Int. J. Environ. Res. Public Health.

[28] Skovdal Rathleff M., Thorborg K., Bandholm T. (2013). Concentric and Eccentric Time-Under-Tension during Strengthening Exercises: Validity and Reliability of Stretch-Sensor Recordings from an Elastic Exercise-Band. PLoS ONE.

[29] Marques D.L., Neiva H.P., Faíl L.B., Gil M.H., Marques M.C. (2019). Acute effects of low and high-volume resistance training on hemodynamic, metabolic and neuromuscular parameters in older adults. Exp. Gerontol..

[30] Hopkins W.G., Marshall S.W., Batterham A.M., Hanin J. (2009). Progressive statistics for studies in sports medicine and exercise science. Med. Sci. Sports Exerc..

[31] Willardson J.M., Burkett L.N. (2006). The effect of rest interval length on the sustainability of squat and bench press repetitions. J. Strength Cond. Res..

[32] Richmond S.R., Godard M.P. (2004). The effects of varied rest periods between sets to failure using the bench press in recreationally trained men. J. Strength Cond. Res..

[33] Willardson J.M., Burkett L.N. (2006). The Effect of Rest Interval Length on Bench Press Performance With Heavy vs. Light Loads. J. Strength Cond. Res..

[34] Rodríguez-Rosell D., Yáñez-García J.M., Mora-Custodio R., Torres-Torrelo J., Ribas-Serna J., González-Badillo J.J. (2020). Role of the Effort Index in Predicting Neuromuscular Fatigue During Resistance Exercises. J. Strength Cond. Res..

[35] Moreira O.C., Faraci L.L., de Matos D.G., Mazini Filho M.L., da Silva S.F., Aidar F.J., Hickner R.C., de Oliveira C.E.P. (2017). Cardiovascular Responses to Unilateral, Bilateral, and Alternating Limb Resistance Exercise Performed Using Different Body Segments. J. Strength Cond. Res..

[36] Rezk C.C., Marrache R.C.B., Tinucci T., Mion D., Forjaz C.L.M. (2006). Post-resistance exercise hypotension, hemodynamics, and heart rate variability: Influence of exercise intensity. Eur. J. Appl. Physiol..

[37] de Tarso Veras Farinatti P., Nakamura F.Y., Polito M.D. (2009). Influence of recovery posture on blood pressure and heart rate after resistance exercises in normotensive subjects. J. Strength Cond. Res..

